# Changes in eGFR in adolescent and young adult inpatients receiving nutritional rehabilitation for a restrictive eating disorder: a five-year clinical audit

**DOI:** 10.1186/s40337-025-01405-9

**Published:** 2025-09-29

**Authors:** Kirsten Thompson, Elizabeth Kumiko Parker, Michael R. Kohn, Anita Stefoska-Needham

**Affiliations:** 1 Nutrition and Dietetics, School of Medical, Indigenous and Health Sciences, Faculty of Science, Medicine and Health, University of Wollongong, Wollongong, NSW 2522 Australia; 2 Diabetes & Endocrinology Ambulatory Care Centre, Westmead Hospital, Westmead, NSW 2145 Australia; 3 Department of Dietetics & Nutrition, Westmead Hospital, Westmead, NSW 2145 Australia; 4 Sydney School of Health Sciences, Faculty of Medicine and Health, The University of Sydney, Sydney, NSW 2006 Australia; 5 Peter Beumont Eating Disorders Service, Sydney Local Health District Mental Health Services, Professor Marie Bashir Centre, Level G, 67–73 Missenden Road, Camperdown, NSW 2050 Australia; 6 Chair in Paediatrics & Child Health, School of Medicine, Western Sydney University, Campbelltown, NSW 2560 Australia; 7 Centre for Research Into AdolescentS’ Health (CRASH), Westmead Hospital, Westmead, NSW 2145 Australia; 8 Department of Paediatrics, Campbelltown Hospital, Campbelltown, NSW 2560 Australia; 9 School of Health Sciences, Faculty of Medicine and Health, University of New South Wales, Sydney, NSW 2052 Australia

**Keywords:** Renal function, Eating disorders, Protein, Nutritional rehabilitation

## Abstract

**Background:**

Impaired renal function may be observed in individuals with a restrictive eating disorder, yet its prevalence and underlying pathophysiological mechanisms are inadequately characterised. The impact of elevated protein intake on estimated glomerular filtration rate (eGFR) in this demographic remains unclear, leading to a lack of specific guidelines regarding protein prescriptions during inpatient treatment. This study describes changes in eGFR as a marker of renal function among adolescents and young adults receiving inpatient care for restrictive eating disorders and evaluates protein prescriptions during nutritional rehabilitation.

**Methods:**

A retrospective audit of adolescent and young adults hospitalised with restrictive eating disorders (2016–2020) on a specialised medical ward was conducted. Data collected included anthropometric measurements, age, serum creatinine, blood urea, energy and protein prescriptions, medical stability upon admission, and hospital length-of-stay. The eGFR was calculated using the CKiDU25 equation. A random intercepts model was employed to assess the relationship between protein intake and eGFR changes during hospitalisation, controlling for confounding variables including age, sex, %mBMI, medical instability, and purging history.

**Results:**

Among the 187 admissions that met inclusion criteria, the mean age was 17.0 ± 1.2 years, 90.9% (*n* = 170) were females, and mean %mBMI was 80.1 ± 9.5% at admission. Impaired renal function (eGFR < 90 mL/min/1.73 m²) was observed in 35.3% of patients at admission, and 3.2% of patients at discharge. Protein intake increased from 1.9 ± 0.4 g/kg/day on admission to 2.7 ± 0.6 g/kg/day at discharge.

**Conclusions:**

Impaired renal function was observed in approximately one third of this sample of adolescents and young adults hospitalised with restrictive eating disorders and typically resolves during the admission. A high protein prescription of 1.9–2.7 g/kg/day did not deleteriously affect renal function, with eGFR levels improving with nutritional rehabilitation. Prospective studies are needed to confirm the optimal protein prescription during nutritional rehabilitation in patients hospitalised with restrictive eating disorders, and to further explore outcomes in the small subgroup of patients who remain with renal impairment at discharge.

**Supplementary Information:**

The online version contains supplementary material available at 10.1186/s40337-025-01405-9.

## Plain language summary

Impaired kidney function is a potential complication of malnutrition in individuals with restrictive eating disorders, although its prevalence is poorly characterised. During refeeding, increased energy intake may lead to high protein consumption, but the impact of this on kidney function, as measured by estimated glomerular filtration rate (eGFR), remains unclear in this population. This study describes changes in eGFR as a marker of kidney function among adolescents and young adults receiving inpatient care for restrictive eating disorders and evaluates protein prescriptions during nutritional rehabilitation. Over a five-year period, 187 patients met the inclusion criteria for this study and were included in the analysis. Of these, 35.3% presented with impaired kidney function at admission, and 3.2% of cases remained with impaired kidney function at discharge. Protein intake increased from 1.9 ± 0.4 g/kg/day at admission to 2.7 ± 0.6 g/kg/day at discharge. Overall, the study suggests that a high protein prescription did not negatively affect kidney function, with eGFR normalised for the majority of patients by discharge. Further prospective studies are needed to determine the optimal protein prescription for patients with restrictive eating disorders during medical refeeding.

**Supplementary Information:**

The online version contains supplementary material available at 10.1186/s40337-025-01405-9.

## Background

Restrictive eating disorders (EDs), including anorexia nervosa (AN), atypical anorexia nervosa (AAN), and avoidant/restrictive food intake disorder (ARFID), are serious illnesses associated with disturbances in food-related thoughts and emotions, and eating behaviours [1, 2]. Restrictive EDs are associated with higher rates of morbidity and mortality [3], and their prevalence is rising, especially amongst young people aged 15–19 years [4]. Poor oral intake, with or without purging behaviours, is a common feature of restrictive EDs and can result in protein–energy malnutrition [5, 6], with medical complications such as electrolyte and hormonal disturbances [7–9], cardiovascular instability including bradycardia [6–8], and impaired renal function [10, 11]. 

Individuals hospitalised with restrictive EDs may present with impaired renal function as a result of prerenal (dehydration), renal (parenchymal changes), or post renal (nephrolithiasis) complications [12, 13]. Pre-renal causes of impaired renal function, such as reduced kidney perfusion and some renal causes (acute tubular necrosis), are generally considered reversible with nutritional rehabilitation [10]. These conditions can result from dehydration, hypovolemia, purging, low cardiac outflow, and rhabdomyolysis [10]. Among adolescents and young adults hospitalised with restrictive EDs, whose illness duration is generally shorter than that of older adults, renal impairment at admission is hypothesised to be predominantly pre-renal and thus expected to improve with nutritional rehabilitation. Structural renal damage from chronic hypokalaemia and diuretic misuse, leading to chronic tubulointerstitial nephritis or nephrocalcinosis, is less commonly observed [10]. Post-renal causes, such as nephrolithiasis, are also infrequent [10]. 

Renal function is commonly assessed using estimated glomerular filtration rate (eGFR) [10], with values < 60mL/min/1.73m^2^ considered clinically significant in adults with chronic kidney disease (CKD) [14]. However, in ED populations, a threshold of < 90mL/min/1.73m^2^ is often used, and this level has been reported in 4–96% of hospitalised patients with restrictive EDs [12, 15–21]. Multiple equations have been used to estimate eGFR in adolescents with EDs, including the Cockroft-Gault, MAYO Clinical Quadratic (MCQ), Chronic Kidney Disease Epidemiology Collaboration (CKD-EPI), the Modification of Diet in Renal Disease (MDRD), and Schwartz [16, 17]. Although the Cockroft-Gault is often recommended for adolescents with EDs [16], its accuracy is inconsistent in malnourished or paediatric populations [22, 23]. The modified Schwartz equation [24] has also been applied [12, 15], but lacks validation for patients over 18 years or those with EDs [13]. The more recent ‘Chronic Kidney Disease in Children Under 25 Years’ (CKiDU25) equation, validated in 2020 for individuals up to 25 years, offers improved accuracy over Schwartz [25], especially in the context of low muscle mass and reduced serum creatinine [25–28]. Since eGFR is strongly associated with metabolic rate and body surface area (BSA), equations typically adjust for BSA to enable inter-individual comparison. However, indexing to standard BSA can overestimate eGFR in malnourished people, potentially masking renal impairment [27]. 

Nutritional rehabilitation, comprising provision of adequate energy, micro- and macronutrient intake, is the first-line inpatient treatment for restrictive EDs [29], aiming to restore weight and correct complications of protein-energy malnutrition [29, 30]. Although impaired renal function is a recognised complication of malnutrition in EDs, the effects of nutritional rehabilitation on renal function remain underexplored [7]. Existing clinical guidelines primarily focus on the prevention and monitoring of refeeding syndrome (RFS), a potentially life-threatening shift in electrolytes and fluids following reintroduction of nutrition [31], however they do not address the potential renal consequences of nutritional rehabilitation [1, 32–36]. 

Nutritional rehabilitation for adolescents and young adults with restrictive EDs typically involves high-protein intake in conjunction with increased energy provision necessary for weight restoration and for reversal of malnutrition-related complications; however, its impact on renal function remains unclear. While high-protein diets are known to affect renal outcomes in CKD generally [14, 37], it is unknown whether similar effects occur in the adolescent and young adult EDs context. The *Clinical Practice Guidelines for the BC Eating Disorders Continuum of Services* recommend protein intakes of 1.0–1.5 g/kg/day for adults, but no equivalent recommendations exist for adolescents [34]. In Australia, national ED guidelines do not include specific recommendations for protein provision in either adults or adolescents and young people [1, 35, 36]. In the absence of specific guidance, general nutrition standards are typically followed in clinical practice, recommending 1.0–1.5 g protein/kg/day for malnourished adults [38, 39], and a recommended daily intake (RDI) of 0.99 g protein/kg/day for adolescents aged 14-18yrs [40]. The limited evidence on renal outcomes during nutritional rehabilitation in adolescents and young adults with EDs may explain the lack of targeted protein guidance.

This study investigated changes in renal function, measured by eGFR using the CKiDU25 equation, in a group of hospitalised adolescents and young adults with restrictive EDs undergoing nutritional rehabilitation, by assessing protein intake levels with changes in eGFR.

## Methods

### Study design and study population

**Fig. 1:**
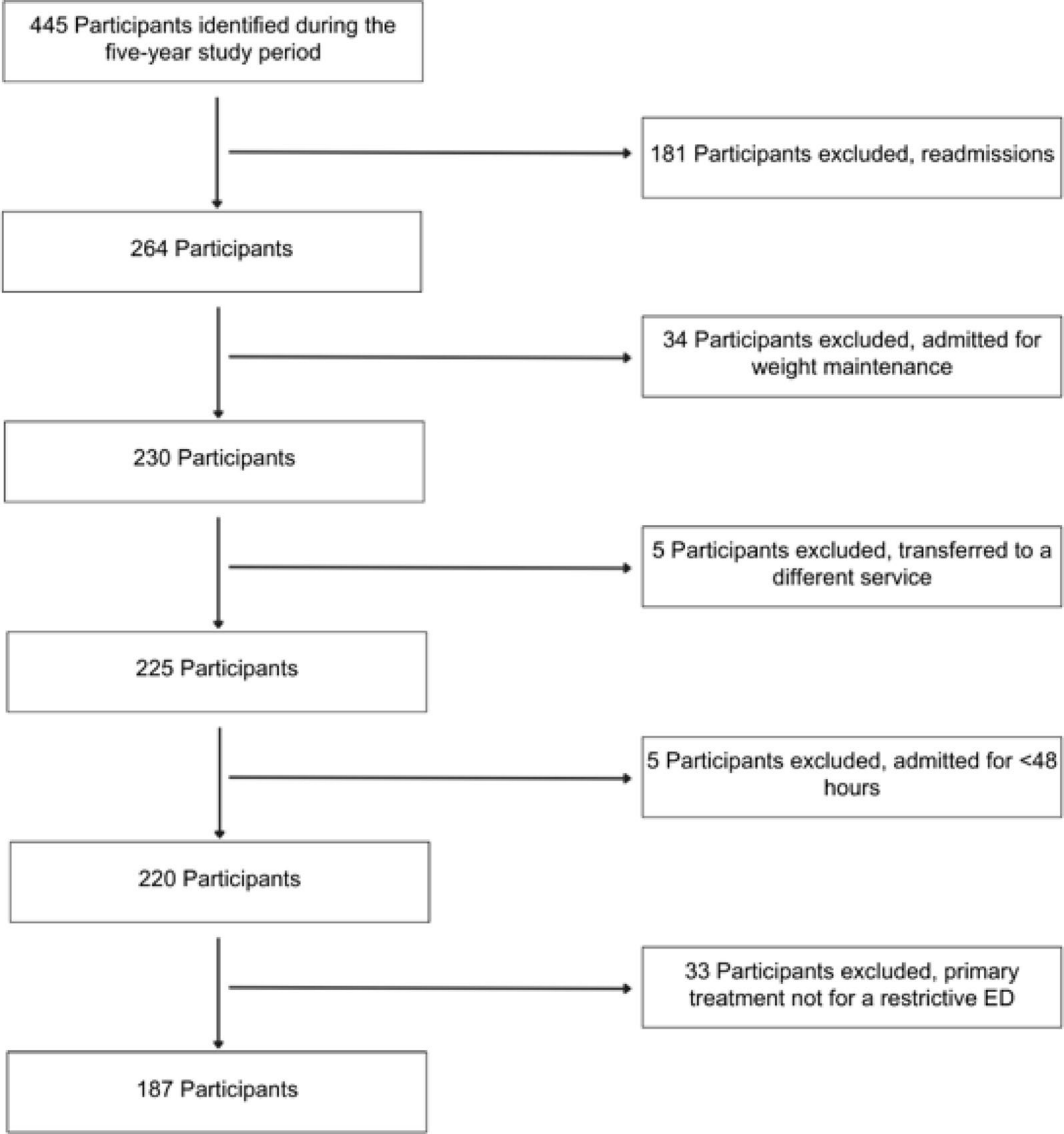
Flow chart of participant recruitment after applying exclusion and inclusion criteria

A retrospective cross-sectional clinical audit of adolescent and young adults admitted with a restrictive ED to a tertiary teaching hospital in metropolitan Sydney, Australia, was conducted between January 2016 and December 2020. Eligible participants included adolescents and young adults who were (i) aged 14–21 years; (ii) diagnosed with a restrictive ED in accordance with the DSM-5 criteria [2] (such as AN, AAN and ARFID); (iii) admitted as an inpatient for > 48 h; and (iv) receiving nutritional rehabilitation as part of their inpatient treatment. Only the first admission during the study period was included. Refer to Fig. 1 for additional information on inclusion/exclusion criteria.

The study was guided by and reported according to the ‘Strengthening the reporting of observational studies in epidemiology (STROBE) checklist’ [41] for cross-sectional studies (Supplementary File 1). This study received ethical approval from Western Sydney Local Health District HREC (reference HREC/14/WMEAD/18). Due to the nature of a retrospective audit, consent was not sought from patients.

## Data collection

Data from paper-based and electronic medical records were collected, including weight, height, age, sex, serum creatinine and blood urea, energy and protein prescription (using actual weight to calculate kcal/kg/day and g/kg/day, respectively), medical stability on admission, history of purging, and hospital length of stay. Percentage median body mass index (%mBMI) was calculated using the CDC clinical growth charts at the 50th percentile BMI for age and sex [42]. The CKiDU25 equation was used to determine eGFR, as an indication of renal function.

CKiDU25 equation: [25]

$${\text{eGFR }}({\text{mL}}/{\text{min}}/{\text{1}}.{\text{73 }}{{\text{m}}^{{\text{2}})}}={\text{ K x Ht}}/{\text{SCr}}$$

where:

K = sex- and age-dependent K value (Supplementary File 2).

SCr = serum creatinine in mg/dL (note: to convert serum creatinine µmol/L to mg/dL, divide by 88.4)

Ht = height in metres.

The eGFR was also calculated using the modified Schwartz equation, and results are presented in Supplementary File 3.

The eGFR was categorised using the description and range: Stage 1: ≥ 90 mL/min/1.73m^2^ (normal or high); Stage 2: 60–89 mL/min/1.73m^2^ (mildly decreased); Stage 3 A: 45–59 mL/min/1.73m^2^ (mildly to moderately decreased); Stage 3B: 30–44 mL/min/1.73m^2^ (moderately to severely decreased); Stage 4: 15–29 mL/min/1.73m^2^ (severely decreased); and Stage 5: < 15 mL/min/1.73m^2^ (kidney failure), in line with the KDIGO working group guidelines [14]. While our cohort did not exhibit chronic renal failure, the eGFR thresholds applied here have been previously used to characterise the degree of renal impairment in individuals with AN [19]. 

### Nutritional rehabilitation

Patients admitted with medical instability, defined as bradycardic (HR ≤ 50 bpm), hypotensive (systolic BP < 80 mm Hg systolic or postural decrease of more than 20 mm Hg systolic), temperature < 35.5 °C, were commenced on continuous nasogastric (NG) feeds with a 1.0 kcal/mL or 1.5 kcal/mL formula providing 1890–2400 kcal/day within 24 h of admission. All patients were prescribed prophylactic phosphate (500 mg twice/day) and a daily multivitamin. Once medically stable, NG feeds were transitioned to nocturnal cyclic (2000–0600 h) and a standardised meal plan was commenced. Meal plans were progressed (1800 kcal, 2300 kcal, 2800 kcal, 3300 kcal, 3800 kcal) as NG feeds were titrated down, and nutrition supplement drinks (300 kcal, 400 kcal) were prescribed in addition to the meal plan if required to meet the minimum weight restoration target of 1 kg/week. Meal support therapy was provided at all meals and mid meals, and a nutrition supplement was provided to patients who were unable to finish 100% of meals or mid meals.

Patients were weighed on three mornings per week (Monday/Wednesday/Friday), before breakfast, after voiding. Regular nursing observations included monitoring of heart rate, respiratory rate, blood pressure, and temperature. Blood tests including serum electrolytes (including potassium, magnesium, phosphate), urea and creatinine, were measured daily during the first week of admission, then decreased to twice weekly, and then weekly when clinically indicated. A weekly medical examination was also performed to assess for delirium, peripheral oedema and other clinical symptoms of RFS. The treating multidisciplinary team reviewed patient progress at meetings three times per week, and decisions were made to adjust NG feed and/or oral meal plan based on clinical progress.

### Data analysis and statistics

Data was collected and analysed using SPSS for Windows Version 26, IBM Corporation. Descriptive statistics were used to describe the population and were reported as mean with standard deviation, median with lower quartile and upper quartile, and number (%) for frequency. A random intercepts model accounting for patient level differences was used to examine how protein affects the eGFR level across the course of the admission, adjusting for age, sex, %mBMI, medical instability and history of purging. Statistical significance was set at p-value < 0.05.

## Results

Of the 445 admissions identified during the five-year study period, a total of 187 admissions met the eligibility criteria and were included in this analysis (Fig. 1). A total of 172 (92%) participants were diagnosed with AN, 14 (7%) ARFID and one (0.5%) with AAN. A total of 130 (70%) patients were medically unstable on admission and 138 (73%) patients engaged in purging behaviours. Of the 130 participants who were admitted with medical instability, 113 (87%) of the participants experienced bradycardia (heart rate ≤ 50 bpm). Most participants were female (*n* = 170, 91%). On admission participants had a mean age of 17 ± 1.2 years, and %mBMI of 80.1 ± 9.5%. A total of 170 (91%) participants received feeding via a nasogastric (NG) tube within the first 48 h of admission. Further demographic data of the study cohort is presented in Table 1.

**Table 1:** Characteristics of adolescent and young adult patients hospitalised with a restrictive eating disorder (*n* = 187)

Variable	Mean (SD)	Median (LQ-UQ)
Age (years)	17.0 (1.2)	16.8 (16.3–17.5)
Admission weight (kg)	45.6 (7.0)	45.0 (41.5–49.2)
Discharge weight (kg)	53.5 (6.4)	52.9 (49.5–56.8)
Admission BMI (kg/m^2^)	16.7 (1.9)	16.7 (15.5–17.8)
Discharge BMI (kg/m^2^)	19.6 (1.4)	19.5 (18.8–20.2)
Admission %mBMI (%)	80.1 (9.5)	80.1 (74.3–85.5)
Discharge %mBMI (%)	93.9 (7.1)	93.6 (90.5–97.2)
Change in %mBMI (%)	13.9 (6.8)	13.6 (8.9–17.2)
Energy Intake on admission		
kcal/day	2219 (461)	1890 (1890–2400)
kcal/kg/day	50 (12)	49 (41–57)
Energy intake on discharge		
kcal/day	3732 (649)	3800 (3300–4200)
kcal/kg/day	71 (15)	71 (62–80)
Protein intake on admission (g/kg/day)	1.9 (0.4)	1.9 (1.7–2.2)
Protein intake on discharge (g/kg/day)	2.7 (0.6)	2.7 (2.3-3.0)
Weight gain per week (kg/week)	2.2 (0.8)	2.2 (1.8–2.6)
Length of hospital stay (days)	26 (13)	23 (17–34)
Maximum creatinine, serum (umol/L)	65.5 (12.9)	64.0 (57–71)

*BMI* Body mass index, *%mBMI* Percentage median body mass index

During the admission, when using the CKiDU25 equation, eGFR increased by 9.70 mL/min/1.73m^2^ for each additional 1 g/kg/day of protein (*p* < 0.001, 95%CI 8.3, 11.2). The effect of age, sex, %mBMI, medical instability on admission, presence of purging, and the effect over time of protein on eGFR are reported in Table 2.

**Table 2:** Correlations between independent variables and eGFR as reported by the CKiDU25 equation (*n* = 187)

Coefficient	Estimate	Std. Error	t value	*p* value	95% CI
Day of admission	0.06	0.04	1.58	0.115	-0.02,0.14
Protein (g/kg/day)	9.70	0.72	13.41	< 0.001	8.3,11.2
Age	2.07	1.22	1.69	0.093	-0.3,4.4
Sex (male)	20.44	5.22	3.91	< 0.001	10.3,30.6
%mBMI	0.64	0.06	10.49	< 0.001	0.5,0.8
Medically unstable	-7.62	3.09	-2.47	0.015	-13.6,-1.6
History of purging	-5.64	3.24	-1.74	0.083	-11.9,0.6
Day of admission : Protein (g/kg)	-0.02	0.01	-2.17	0.030	-0.048,-0.003

*%mBMI* Percentage median body mass index

Impaired renal function, defined as eGFR < 90 mL/min/1.73m^2^, was present in 35.3% (*n* = 66) of patients on admission, and in 3.2% (*n* = 6) on discharge, indicating the majority of cases of renal impairment resolved with nutritional rehabilitation (Table 3). The small number of patients discharged with impaired renal function precluded further statistical analysis; however, within this subgroup (*n* = 6), eGFR at discharge ranged from 73.2 to 86.1 mL/min/1.73 m². Results using the modified Schwartz equation [24] are presented in Supplementary File 3.

**Table 3:** Number (%) of patients with normal (Stage 1) or impaired eGFR (Stages 2–5) on admission and discharge using CKiDU25 equation (n *=* 187)

	eGFR on Admission	eGFR on Discharge
Stage 1: ≥ 90 mL/min/1.73m^2^	121 (64.7%)	181 (96.8%)
Stage 2: 60–89 mL/min/1.73m^2^	64 (34.2%)	6 (3.2%)
Stage 3 A: 45–59 mL/min/1.73m^2^	2 (1.1%)	0
Stage 3B: 30–44 mL/min/1.73m^2^	0	0
Stage 4: 15–29 mL/min/1.73m^2^	0	0
Stage 5: < 15 mL/min/1.73m^2^	0	0

## Discussion

This study assessed changes in eGFR in a sample of adolescent and young adults hospitalised with a restrictive ED during nutritional rehabilitation. Protein effects on eGFR during admission were investigated after adjusting for age, sex, %mBMI, medical instability and history of purging. At admission, approximately one third of patients (35.3%) presented to hospital with impaired renal function (defined as < 90 mL/min/1.73m^2^) and received an average protein intake of 1.9 ± 0.4 g/kg/day as part of nutritional rehabilitation. By discharge, protein provision had increased to 2.7 ± 0.6 g/kg/day on average. Renal function normalised during admission in most cases, with 96.8% of patients discharged with eGFR ≥ 90 mL/min/1.73m^2^. These findings are notable given concerns about high-protein intake affecting renal function indicators such as eGFR [43]. 

While the current study aligns with previous research identifying the presence of renal impairment in adolescents with restrictive EDs, meaningful cross-study comparisons are limited by methodological variability and variations in validity. For example, the current study reported a 35.3% prevalence of impaired renal function on admission using the CKiDU25 equation, which was similar to the 33% and 36.8% reported by Downey et al. (2022) and Gurevich et al. (2021), respectively, who used the modified Schwartz equation in cohorts of adolescents hospitalised with AN [12, 15]. The modified Schwartz equation, although commonly used, is not validated for individuals over 18 years of age. In contrast, the CKiDU25 equation, developed by the same research team, was used in the current study and has the advantage of being validated for patients up to 25 years old. However, neither equation has been specifically validated for the malnourished patients with restrictive EDs, and a limitation of the current study is the absence of isotopic measurements to confirm the accuracy of the CKiDU25 equation in this population.

The choice of eGFR estimation method appears to significantly influence prevalence estimates, as demonstrated in a Canadian study of 51 adolescents hospitalised with AN [17]. Renal impairment on admission varied greatly depending on the equation used to calculate eGFR: 45% (Cockcroft-Gault), 28% (MDRD), 14% (CKD-EPI), 12% (MCQ), and 4% (Schwartz)[17]. Studies conducted in France, such as those by Mignot-Bedetti et al. (2018) and Stheneur et al. (2024), reported higher prevalence (96% and 82%, respectively) of impaired renal function in adolescents hospitalised with AN using the Cockcroft-Gault Eqs. [19, 20] A study involving 34 adolescents hospitalised with AN in Canada found that the Cockcroft-Gault equation had the best correlation with GFR measured by isotopic technique [16]. The main advantage of the Cockcroft-Gault equation is that it is the only equation that considers the patient’s weight [18]. However, others studies have reported this equation may be inaccurate when applied to malnourished patients [22], or children aged 1–18 years [23]. The variation in reported prevalence rates is likely due to methodological differences across studies, highlighting the need for standardised, population-appropriate methods for estimating renal function in adolescents and young adults with restrictive EDs.

Creatinine-based equations for estimating GFR offer the benefit of being calculated from a single blood test. However, their main limitation is that low muscle mass and low dietary protein intake can affect creatinine levels, leading to an overestimation of eGFR in malnourished patients [27, 44]. Furthermore, accuracy and validity of some equations decrease with age, particularly after 18 years, owing to the absence of an age-dependent constant [45]. The current study addresses this limitation by using the CKiDU25 equation, validated for individuals up to age 25, which incorporates age- and sex-dependent constants, improving the accuracy of GFR estimation [25]. 

In the current study, patients with a history of purging behaviours did not have significantly more renal damage than those who did not purge. While this result may be somewhat unexpected, given the established pathophysiological links between purging, hypovolaemia, and electrolyte disturbances, particularly hypokalaemia [12, 46], the findings are in line with other studies which have reported medical instability (including bradycardia) [12], [15] and degree of malnutrition (e.g. BMI or %mBMI) [18, 19] as the primary factors correlated with reduced eGFR. In a study of 106 adolescent girls hospitalised with AN in France, Mignot-Bedetti et al. (2018) reported no difference in creatinine clearance rate between binge/purge versus restrictive subtypes on admission, however at discharge, patients with the binge/purge subtype had a significantly lower creatinine clearance rate compared with patients with a restrictive subtype (75.8 mL/min versus 86.7 mL/min, *p* < 0.01) [20]. Further investigations are needed, which incorporate more precise measures such as serum potassium levels, detailed fluid balance assessments, and frequency or intensity of purging episodes to better understand potential mechanisms and clinical implications in this subgroup.

In a retrospective case-controlled study by Gurevich et al. (2021) of 395 adolescents hospitalised with AN in Israel, renal impairment was reported in 36.8% of patients using the modified Schwartz equation, which transiently worsened during the admission [12]. The authors postulated an undefined mechanism, independent of dehydration, caused renal impairment in these patients, claiming adequate hydration was maintained during hospitalisation [12], and acknowledged they could not collect data on the subtype of ED due to the retrospective nature of the study. A limitation of the current study is the lack of urine analysis, making it unclear whether the observed impaired renal function in this patient subset was due to protein energy malnutrition or prerenal causes such as dehydration. Acknowledging these limitations is crucial for understanding the study’s scope, the generalisability of its findings, and their potential implications for future research and clinical practice.

To the authors’ knowledge, this is the first study to report on protein intake in relation to renal function as measured by eGFR, in adolescents and young adults with restrictive EDs. The average intake of protein on day one of admission was 1.9 ± 0.4 g/kg/day, which is double the RDI for hospitalised adolescents based on recommendations in the *ACI Nutrition Standards* [40]. Upon discharge the average intake of protein increased to 2.7 ± 0.6 g/kg/day, and the majority of cases of impaired renal function had resolved, indicating that the high protein intake was not associated with deleterious effects on renal function. This finding is important as it suggests that high protein intakes during higher-energy nutritional rehabilitation in this patient group did not appear to be associated with deleterious consequences on renal function. However, the results of this study should be interpreted with caution, as their broader generalisability remains unclear and requires confirmation through prospective studies. A further strength of this study is its five-year data collection period, which enabled a comprehensive analysis of trends and patterns, contributing to a more robust understanding of higher-energy nutritional rehabilitation and renal impairment. Notably, a small subgroup of patients (3.2%) were discharged with impaired renal function, despite receiving nutritional rehabilitation, highlighting the need for closer monitoring and further investigation. Further research is also needed to identify factors that may contribute to longer-term impaired renal function in this patient population, such as prolonged duration of illness potentially contributing to structural kidney changes. Due to the retrospective nature of the current study, data on the duration of illness were not available in this study.

While contributing valuable insights, the study is not without its limitations. This single-site study employed a retrospective study design, which inherently carries the risk of selection bias and limited control over the quality of data collected. Furthermore, the reliance on both paper and electronic records introduced challenges related to accuracy and validity, given the inherent risk of incomplete documentation. Additionally, the study included only 42% of total admissions during the study period, as it focused exclusively on patients requiring nutritional rehabilitation on only the first admission during the study period.

The findings of this study are important, as patients with restrictive EDs and reduced eGFR levels have a significant risk of developing chronic kidney disease over their lifetime [12, 46]. Future prospective studies are recommended, and should include additional comprehensive assessments and continuous monitoring, which could encompass urinalysis, evaluations of urine protein-to-creatinine ratios, and renal ultrasounds [15, 18]. Furthermore, this study highlights the need for more extensive multicentre studies to validate the findings across diverse healthcare settings, ultimately contributing to a broader and more generalisable body of knowledge in the field of restrictive EDs and impaired renal function.

## Conclusion

Impaired renal function occurred in approximately one third of adolescent and young adults hospitalised with a restrictive ED. A high protein prescription did not deleteriously affect renal function, with eGFR levels normalising for the majority of patients by discharge. In the absence of formalised protein guidelines specific to this patient population, the present study provides valuable insights regarding the safety of high protein intakes during higher-energy nutritional rehabilitation. Further evidence from prospective studies is needed to confirm the optimal protein prescription during nutritional rehabilitation in patients hospitalised with restrictive EDs, and to identify the causes of impaired renal function within this patient group. Additionally, long-term follow-up studies are required to evaluate the outcomes of the subset of patients discharged with unresolved renal impairment.

## Supplementary Information

Below is the link to the electronic supplementary material.

Supplementary Material 1

Supplementary Material 2

Supplementary Material 3

## Data Availability

The datasets used and/or analysed during the current study are available from the corresponding author on reasonable request.

## Abbreviations

%mBMI: Percent median body mass index
ACI: Agency for clinical innovation
AN: Anorexia nervosa
AAN: Atypical anorexia nervosa
ARFID: Avoidant/restrictive food intake disorder
BMI: Body mass index
BSA: Body surface area
CKD: Chronic kidney disease
CKiDU25: Chronic kidney disease in children under 25 years
ED: Eating disorder
eGFR: Estimated glomerular filtration rate
KDIGO: Kidney disease improving global outcomes
NG: Nasogastric
RFS: Refeeding syndrome
