# Supplementary material for: Changes in eGFR in adolescent and young adult inpatients receiving nutritional rehabilitation for a restrictive eating disorder: a five-year clinical audit
Source: J Eat Disord. 2025 Sep 29;13:213. doi: 10.1186/s40337-025-01405-9 (PMC12482035; doi:10.1186/s40337-025-01405-9)
Supplement: Supplementary file 2 — Supplementary Material 2 [file 40337_2025_1405_MOESM2_ESM.docx]

Supplementary File 2. Sex- and age-dependent values of *K* for CKiD U25 eGFRcr

| **Age, years** | **Female** | **Male** |
| --- | --- | --- |
| 1 to <12 | 36.1 × 1.008^(Age–12)^ | 39.0 × 1.008^(Age–12)^ |
| 12 to <18 | 36.1 × 1.023^(Age–12)^ | 39.0 × 1.045^(Age–12)^ |
| 18 to 25 | 41.4 | 50.8 |

GFR conversion factors

- GFR mL/min/1.73 m^2^ to mL/s/1.73 m^2^, multiply by 0.0167
- serum creatinine µmol/L to mg/dL, divide by 88.4

Source: https://www.niddk.nih.gov/research-funding/research-programs/kidney-clinical-research-epidemiology/laboratory/glomerular-filtration-rate-equations/children-adolescents-young-adults#ckid-u25
