# Supplementary material for: Changes in eGFR in adolescent and young adult inpatients receiving nutritional rehabilitation for a restrictive eating disorder: a five-year clinical audit
Source: J Eat Disord. 2025 Sep 29;13:213. doi: 10.1186/s40337-025-01405-9 (PMC12482035; doi:10.1186/s40337-025-01405-9)
Supplement: Supplementary file 3 — Supplementary Material 3 [file 40337_2025_1405_MOESM3_ESM.docx]

**Supplementary File 3.** Results of eGFR using modified Schwartz equation

**Table S3:** Number (%) of patients with normal (Stage 1) or impaired eGFR (Stages 2-5) on admission and discharge (n *=* 187)

|  |  |  |
| --- | --- | --- |
|  | Modified Schwartz | Modified Schwartz |
| Stage 1: ≥ 90 mL/min/1.73m^2^ | 127 (67.9%) | 181 (96.8%) |
| Stage 2: 60-89 mL/min/1.73m^2^ | 58 (31.0%) | 6 (3.2%) |
| Stage 3A: 45-59 mL/min/1.73m^2^ | 2 (1.1%) | 0 |
| Stage 3B: 30-44 mL/min/1.73m^2^ | 0 | 0 |
| Stage 4: 15-29 mL/min/1.73m^2^ | 0 | 0 |
| Stage 5: < 15 mL/min/1.73m^2^ | 0 | 0 |

Modified Schwartz equation

eGFR = 0.413 x [height (cm) / serum creatinine (mg/dL)

(note: to convert serum creatinine µmol/L to mg/dL, divide by 88.4)
